# Tumor treating fields suppress tumor cell growth and induce immunogenic cell death biomarkers in biliary tract cancer cell lines

**DOI:** 10.1038/s41598-025-16341-6

**Published:** 2025-08-20

**Authors:** Ying Yue, Yingying Wang, Jingjing Feng, Min Yao, Yuanzhen Suo

**Affiliations:** 1 Healthy Life Innovation Medical Technology Co., Ltd, Wuxi, 214174 China; 2 Liangzhu Laboratory, Zhejiang University, Hangzhou, 310058 China

**Keywords:** Biliary tract cancer, Tumor treating fields, Immunogenic cell death, Cancer therapy, Cancer, Biliary tract cancer

## Abstract

Tumor Treating Fields (TTFields) are low-intensity, intermediate-frequency alternating electric fields that exert antimitotic effects on cancer cells. This study is the first to evaluate the in vitro efficacy of TTFields on biliary tract cancer (BTC) cell lines HCCC-9810 and RBE, investigating their sensitivity to TTFields across varying frequencies and electric field intensities (100–200 kHz, 1.3–2.1 V/cm). The results demonstrated that a frequency of 150 kHz induced the most pronounced cytotoxic effects, significantly impairing clonogenicity and migratory capacity while inducing multipolar spindles and microtubule disorganization. Furthermore, TTFields treatment triggered a marked upregulation of immunogenic cell death (ICD) biomarkers, including enhanced surface exposure of calreticulin (CRT) and increased extracellular release of high mobility group box 1 (HMGB1) and Adenosine triphosphate (ATP). These findings suggest that TTFields hold great promise as a therapeutic strategy for BTC. They not only suppress tumor cell proliferation but also promote the release of ICD-associated biomarkers. Nevertheless, this study is limited to preliminary in vitro experiments. To provide insights for the ongoing clinical trial (NCT06611345), further in vivo studies and mechanistic explorations are essential to investigate the potential of combining TTFields with immune checkpoint inhibitor (ICI) therapy in the treatment of BTC.

**Supplementary Information:**

The online version contains supplementary material available at 10.1038/s41598-025-16341-6.

## Introduction

Biliary tract cancer (BTC), encompassing intrahepatic, perihilar, and distal cholangiocarcinoma as well as gallbladder cancer, represents a heterogeneous group of malignancies with limited treatment options and a generally poor prognosis^1^. The incidence and mortality rates of BTC are rising globally, posing significant challenges in its clinical management^2,3^. Standard therapeutic approaches, such as surgical resection, chemotherapy, and radiation therapy, are often inadequate due to the aggressive nature of the disease, the anatomical complexity of BTC, and the compromised performance status of patients^4,5^. In recent years, targeted therapies and immunotherapies have provided new strategies for treating biliary tract cancers^6,7^. The TOPAZ-1 clinical trial (NCT03875235) represents a significant advancement in immunotherapy^8^. This Phase III study assessed the efficacy of combining durvalumab, an anti-PD-L1 antibody, with the standard gemcitabine and cisplatin (GC) regimen. The trial demonstrated improved overall and progression-free survival (PFS) in patients with BTC who received the durvalumab and GC combination therapy, leading to its approval as a first-line treatment for advanced BTC. However, the efficacy of these treatments remains limited by tumor resistance mechanisms and the immunosuppressive tumor microenvironment.

Tumor Treating Fields (TTFields) represent a novel non-invasive cancer treatment modality that inhibits tumor growth by applying low-intensity (1–3 V/cm), intermediate-frequency (100–300 kHz) alternating electric fields directly to the tumor site. These electric fields are administered to the affected area through transducer arrays positioned on the skin, thereby delivering a localized treatment effect^9,10^. The anticancer effects of TTFields primarily stem from the disruption of mitotic spindle formation during cell division, which results in abnormal chromosome segregation and various types of cell death^11–13^. The landmark EF-14 trial (NCT00916409) showed significant survival benefits in newly diagnosed glioblastoma (GBM) when TTFields were combined with temozolomide, while the STELLAR trial (NCT02397928) established efficacy in malignant pleural mesothelioma, resulting in U.S. Food and Drug Administration (FDA) approval for both indications^14,15^. Currently, TTFields are being investigated in additional solid tumors, including pancreatic cancer, ovarian cancer, brain metastases from non-small cell lung cancer (NSCLC), first-line treatment of NSCLC, and hepatocellular carcinoma^16–20^. These ongoing studies evaluate TTFields in combination with standard-of-care therapies to expand their clinical applications. Preclinical studies demonstrate that the combination of TTFields and ICIs synergistically amplifies antitumor immunity, resulting in improved tumor control and survival outcomes^20,21^. We are conducting a single-arm, Phase 1b clinical trial (NCT06611345) designed to evaluate the therapeutic efficacy and safety of TTFields in combination with the TOPAZ-1 regimen as first-line treatment for patients with unresectable BTC. This investigation constitutes the first registered clinical trial to explore the application of TTFields technology in BTC management.

This study is preclinical and foundational for NCT06611345, represents the first investigation of TTFields applied to BTC cell lines. We systematically evaluated the effects of TTFields on cellular proliferation, migration, mitotic processes, and the expression of ICD biomarkers. We hypothesized that (1) TTFields would suppress BTC proliferation in a frequency- and intensity-dependent manner, as measured by cell count and colony-forming assays. (2) TTFields would inhibit migration of BTC cells, as assessed through scratch assay analysis. (3) TTFields would induce aberrant mitosis in BTC cells, as demonstrated by fluorescent staining of tubulin. (4) TTFields would induce ICD biomarkers in BTC cells, as quantified by flow cytometry and enzyme-linked immunosorbent assay (ELISA).

## Results

### Validation of frequency and intensity of TTFields for human BTC cells

**Fig. 1:**
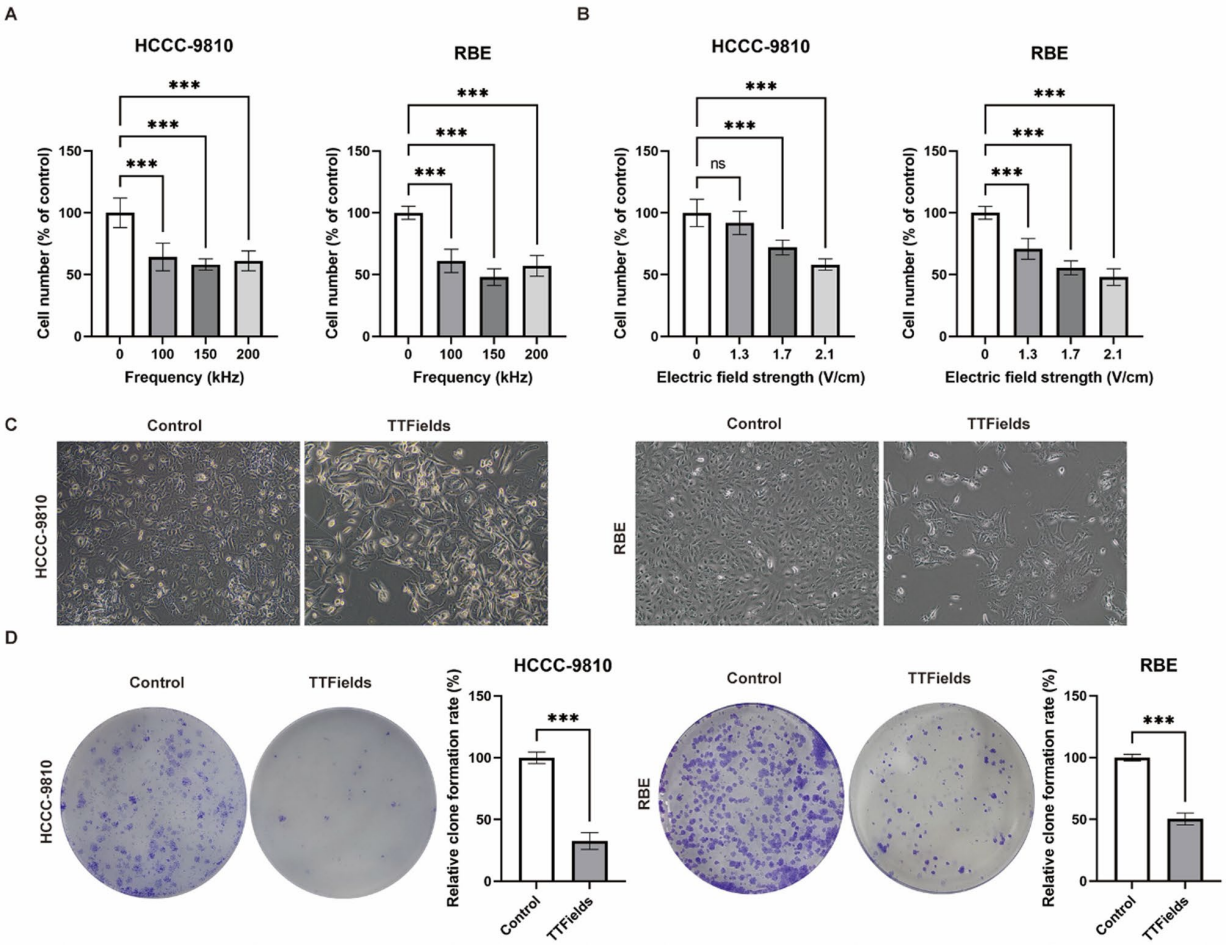
In vitro efficacy of TTFields in human BTC cells. (**A**) HCCC-9810 and RBE cells were treated with TTFields (2.1 V/cm) at different frequencies for 96 h and cell counts were determined (*n* = 6, ***p < 0.001). (**B**) HCCC-9810 and RBE cells were treated with TTFields (150 kHz) at different electric field intensities for 96 h and cell counts were determined (*n* = 6, ns *p* > 0.05, ***p < 0.001). (**C**) Representative images of HCCC-9810 and RBE cell morphology after 96 h of treatment with TTFields (2.1 V/cm). (**D**) Clonogenic potential of HCCC-9810 and RBE cell lines following exposure to TTFields at 2.1 V/cm (*n* = 3, ***p < 0.001).

TTFields exhibit potent antitumor activity across a frequency range of 100–300 kHz, with optimal frequencies being cell type-dependent^9,10^. Consequently, TTFields (2.1 V/cm) at three representative frequencies—100, 150, and 200 kHz—were applied to two BTC cell lines, HCCC-9810 and RBE cells, for a duration of 96 h to ascertain the cellular responses to these different frequencies. The frequency evaluation demonstrated that TTFields treatment significantly suppressed cell proliferation compared to untreated controls across all tested frequencies. While no statistically significant differences were observed among the three frequencies, 150 kHz showed the most pronounced suppressive effect on both BTC cell lines. After 96 h of TTFields treatment, the cell numbers of HCCC9810 and RBE cells were 58.2% (*p* < 0.001) and 48.0% (*p* < 0.001) of those in the control group, respectively (Fig. 1A). Based on these findings, all subsequent experiments were conducted using a frequency of 150 kHz.

The relationship between TTFields intensity and BTC cell proliferation was evaluated at 150 kHz. BTC cells were subjected to TTFields at intensities of 1.3, 1.7, or 2.1 V/cm for 96 h. Significant reductions in cell numbers were observed at 1.7 V/cm for HCCC-9810 (72.0% of control, *p* < 0.001) and 1.3 V/cm for RBE (70.8% of control, *p* < 0.001). Furthermore, a dose-dependent relationship was observed, with cell proliferation decreasing as electric field strength increased (Fig. 1B, Supplementary Fig. 1). Microscopic examination revealed that the cell density was significantly reduced after TTFields treatment, with changes in cell morphology, an increase in cell size, indistinct cell boundaries, and a higher ratio of non-viable cells stained with trypan blue (Fig. 1C, Supplementary Fig. 2). Additionally, the colony-forming capacity of surviving cells decreased significantly after 96 h of TTFields exposure (2.1 V/cm) to 32.6% (*p* < 0.001) of control for HCCC-9810 cells and to 50.4% (*p* < 0.001) of control for RBE cells (Fig. 1D). Collectively, these results demonstrate that TTFields effectively suppress the proliferation of BTC cells.

## TTFields inhibits migration of BTC cells

**Fig. 2:**
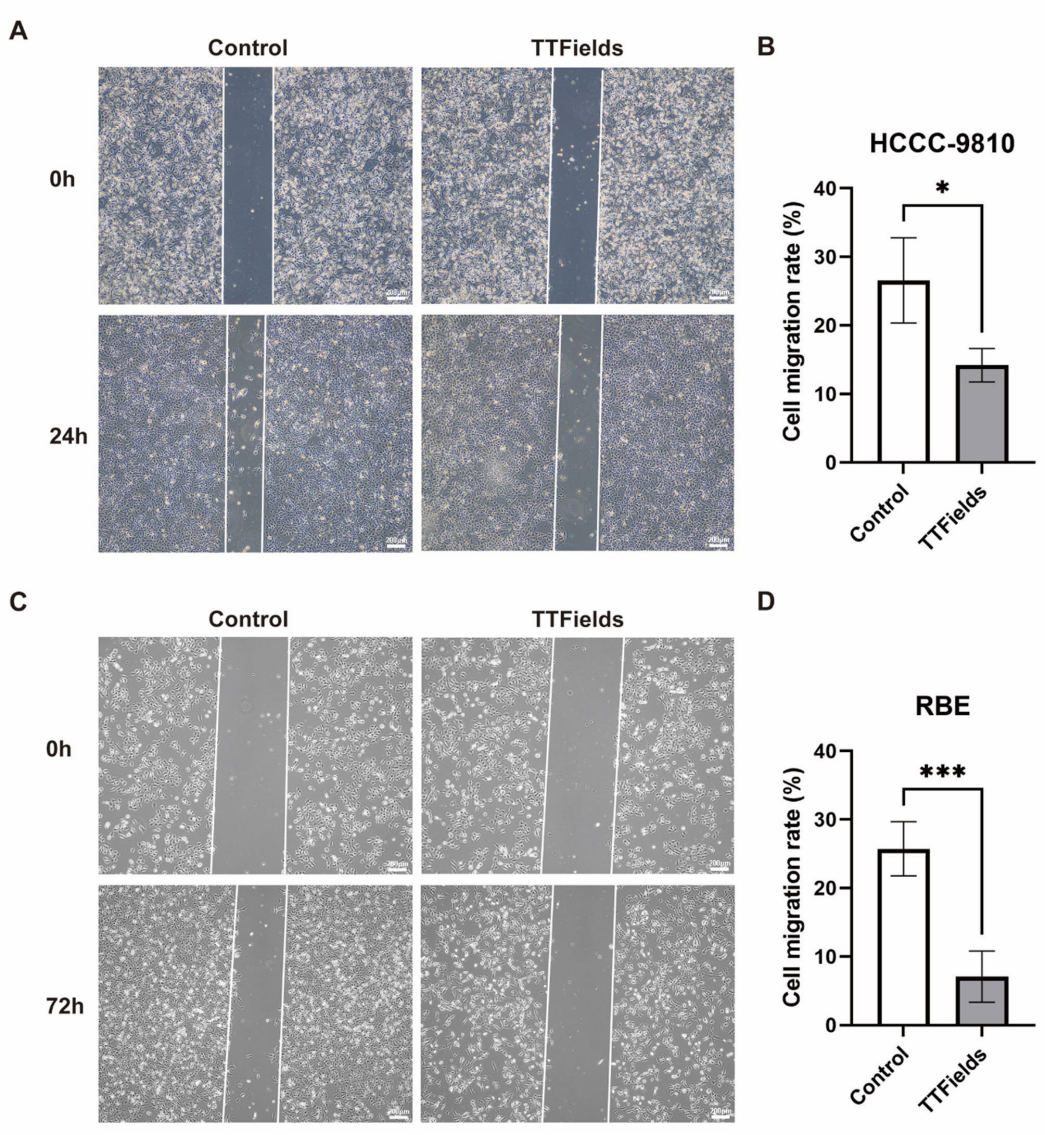
Effect of TTFields on BTC cells migration. (**A**) Representative images of wound healing assay of HCCC-9810 cells in different treatment groups at 0 and 24 h (40×, Scale bar: 200 μm). (**B**) Quantitative data graph of wound healing assay of HCCC-9810 cells. Cellular migration area was quantified following 24 h exposure to TTFields at an intensity of 2.1 V/cm (*n* = 4, *p < 0.05). (**C**) Typical images of the wound healing assay at 0 and 72 h for different treatment groups of RBE cells (40×, Scale bar: 200 μm). (D) Plot of quantitative data of the wound healing assay of RBE cells. Cellular migration area was quantified following 72 h exposure to TTFields at an intensity of 2.1 V/cm (*n* = 6, ***p < 0.001).

Collective migration of tumor cells is the basis of cancer invasion^22,23^. To test the effect of TTFields treatment on the horizontal migratory capacity of BTC cells, we performed wound healing experiments. Typical wound images showed that the wound area of HCCC-9810 and RBE cells was reduced after TTFields (2.1 V/cm) treatment (Fig. 2A, C). Quantitative analysis revealed that TTFields treatment significantly impaired cell migration in both cell lines compared to untreated controls. In HCCC-9810 cells, 24 h of TTFields exposure reduced the migration rate from 26.6 to 14.2% (*p* = 0.010) (Fig. 2B). Similarly, RBE cells demonstrated marked inhibition of migratory capacity following prolonged treatment. After 72 h of TTFields exposure, the migration rate of RBE cells decreased substantially from 25.7 to 7.1% (*p* < 0.001) compared to untreated controls (Fig. 2D). These results suggest that TTFields suppresses BTC cells migration.

## TTFields treatment induces aberrant mitosis in BTC cells

**Fig. 3:**
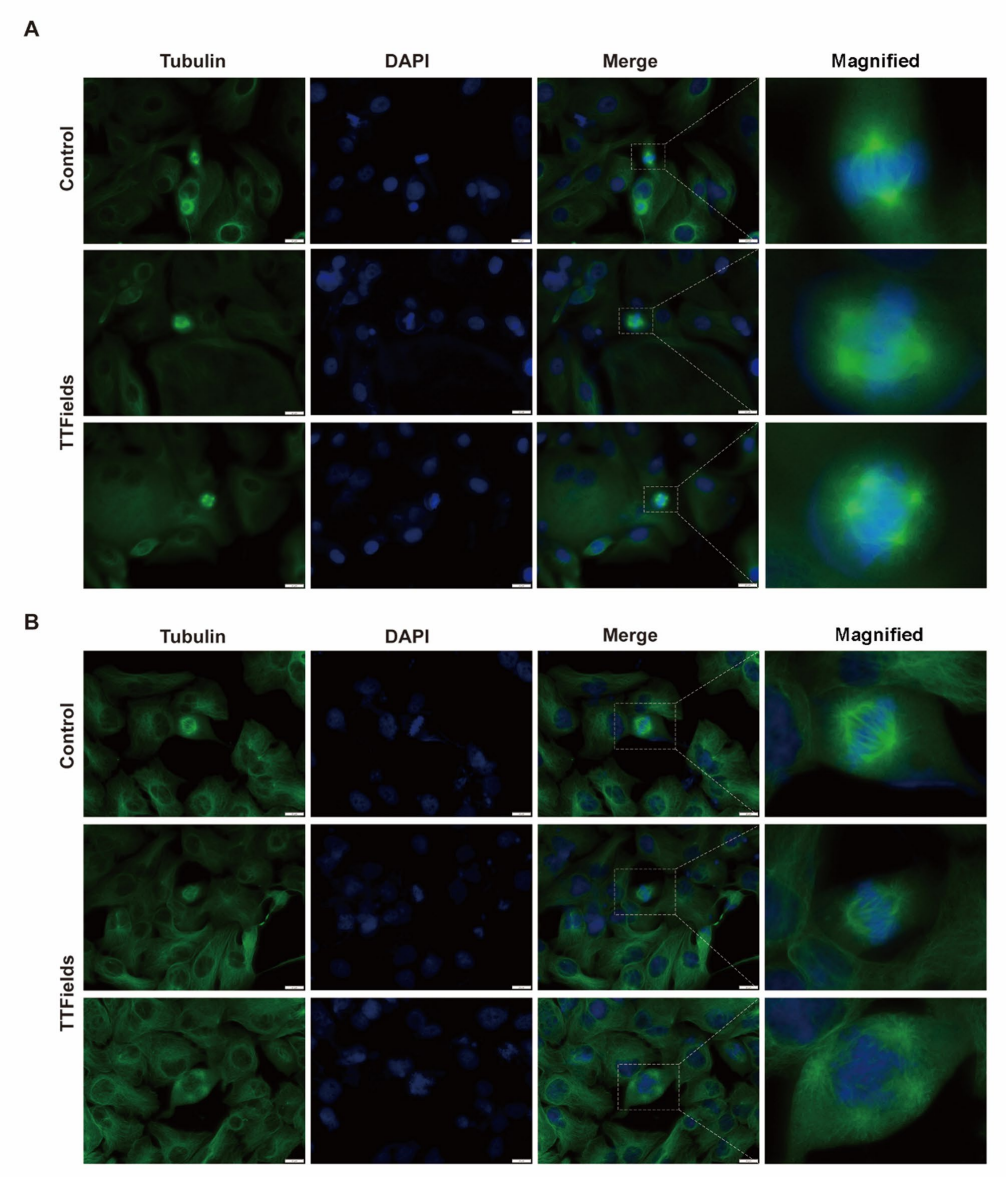
TTFields interfere with spindle morphology in mitosis of BTC cells. BTC cells were treated at 1.7 V/cm for 96 h and then stained with monoclonal antibodies for tubulin (green) and DAPI for DNA (blue), respectively. (**A**) Spindle morphology of HCCC-9810 cells (600×, Scale bar: 20 μm). (**B**) Spindle morphology of RBE cells (600×, Scale bar: 20 μm).

TTFields have been shown to disrupt the normal assembly of the mitotic spindle, thereby interfering with the replication of cancer cells^9,24^. To investigate whether TTFields affect normal mitosis in BTC cells, HCCC-9810 and RBE cell lines were treated with TTFields (1.7 V/cm) for 96 h. Fluorescent staining of tubulin was performed to observe changes in the spindle morphology of dividing cells. The results presented in Fig. 3 indicate that both HCCC-9810 (Fig. 3A) and RBE (Fig. 3B) cells treated with TTFields exhibit abnormal mitosis, including disorganized spindle filament alignment (Upper panel) and multipolar mitosis (Lower panel). This suggests that TTFields treatment interferes with the normal progression of mitosis in the BTC cell line.

## TTFields induces ICD biomarkers in BTC cells

**Fig. 4:**
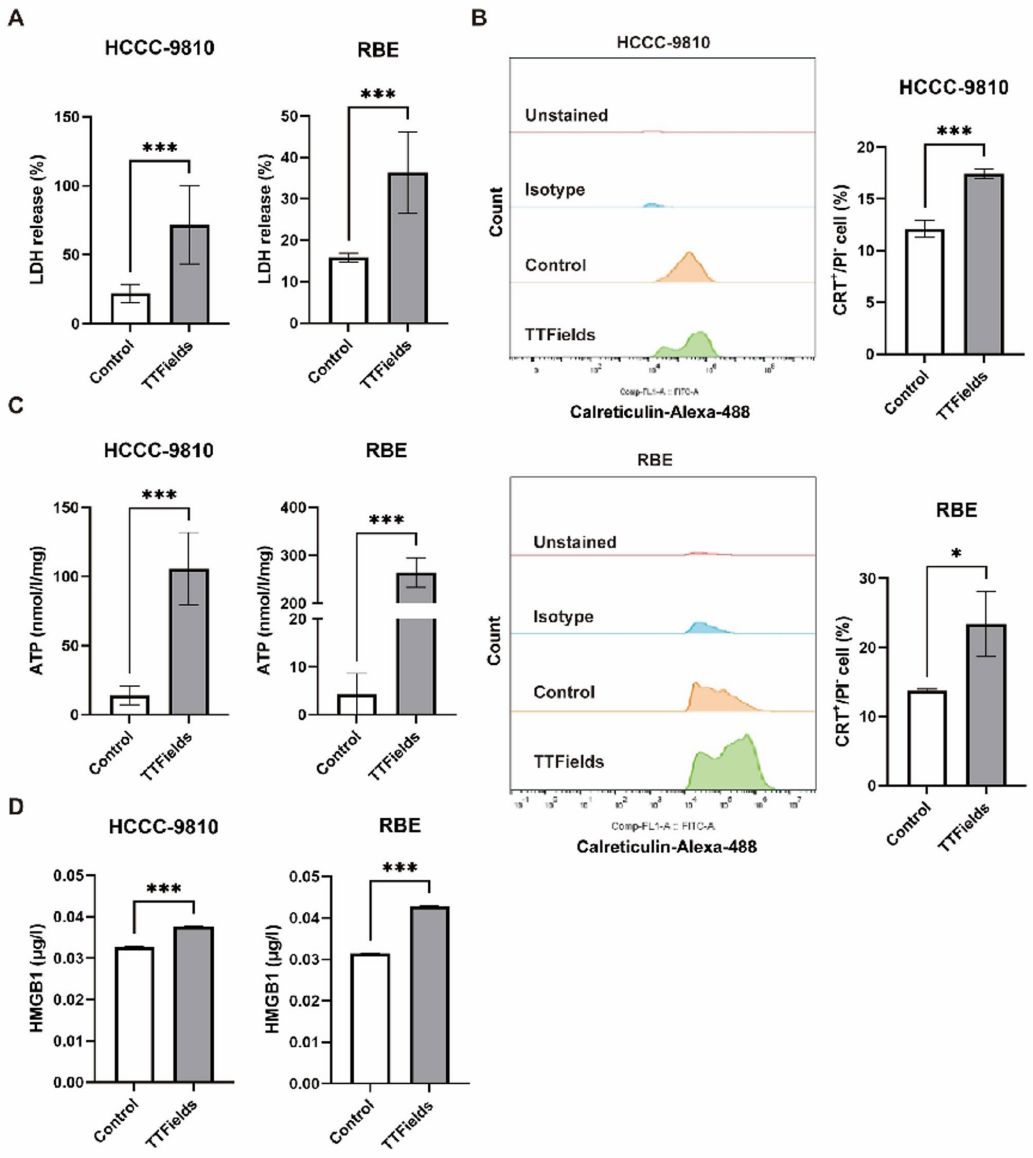
TTFields application promotes DAMPs expression on BTC cells. Conditioned media from cells treated with control or 2.1 V/cm TTFields for 96 h were collected and assayed for biochemical markers of ICD. (**A**) Determination of LDH release in BTC cells after TTFields treatment (*n* = 5, ***p < 0.001). (**B**) Quantitative analysis of CRT surface exposure in live cells (PI-) was performed using a flow cytometer (*n* = 3, *p < 0.05,***p < 0.001). (**C**) Extracellular ATP in the culture medium of BTC cells was detected by fluorometric assay (*n* = 6, ***p < 0.001). (**D**) Soluble HMGB1 in the culture medium of BTC cells was detected by ELISA kit (*n* = 6, ***p < 0.001).

Following 96 h of TTFields treatment, HCCC-9810 and RBE cells exhibited significant cytotoxicity as evidenced by increased extracellular Lactate dehydrogenase (LDH) release. Specifically, LDH release increased from 21.9% in control HCCC-9810 cells to 71.7% in TTFields-treated cells, representing a 3.3-fold increase (*p* < 0.001). Similarly, RBE cells showed an increase in LDH release from 15.8% in controls to 36.3% following TTFields treatment, corresponding to a 2.3-fold elevation (*p* < 0.001) (Fig. 4A). These LDH results directly indicate that TTFields treatment leads to substantial BTC cell damage or death. ICD represents a distinct mode of cell death that elicits an immune response against dead-cell-associated antigens, particularly when released by cancer cells^25–27^. Previous research has established that TTFields can trigger ICD in various cancer cells^21^. To evaluate whether TTFields application induces ICD in BTC cells, HCCC-9810 and RBE cell lines were exposed to 2.1 V/cm TTFields for 96 h. Subsequently, several damage-associated molecular patterns (DAMPs) —established biomarkers of ICD—were assessed, including CRT exposure on the cell membrane, and extracellular secretion of ATP and HMGB1^28^. We first examined the translocation of the chaperone CRT to the cell surface of cells. Using flow cytometric analysis, we found that TTFields treatment significantly boosted the presence of CRT on the surface of viable BTC cell lines (PI^−^). Specifically, the percentage of CRT-positive HCCC-9810 cells rose from 12.1% in control groups to 17.4% after TTFields treatment (*p* < 0.001). Likewise, RBE cells showed a similar increase in CRT-positive cells, going from 13.7% in controls to 23.4% following TTFields exposure (*p* < 0.05) (Fig. 4B, Supplementary Fig. 3). Furthermore, after 96 h of TTFields exposure, we observed a significant increase in ATP levels in the supernatant of both HCCC-9810 and RBE cells (*p* < 0.001 for both) (Fig. 4C). In parallel, extracellular HMGB1 levels also climbed significantly in TTFields-treated HCCC-9810 and RBE cells (*p* < 0.001 for both) (Fig. 4D). Collectively, these findings suggest that TTFields treatment effectively induces multiple ICD biomarkers in BTC cell lines.

## Discussion

In this study, we provide the first evidence that TTFields suppress tumor cell growth and induce ICD biomarkers in BTC cells. We determined that a frequency of 150 kHz produces optimal cytotoxic effects when TTFields are applied to HCCC-9810 and RBE human BTC cell lines, and confirmed that TTFields efficacy is intensity-dependent. Beyond cytotoxic effects, TTFields treatment significantly suppressed both colony formation and cell migration in BTC cells. Furthermore, TTFields induced characteristic ICD biomarkers, evidenced by enhanced surface expression of CRT, as well as elevated secretion of HMGB1 and ATP into the conditioned medium.

TTFields exert direct effects on mitotic division in BTC cells through their alternating electric fields, as illustrated in Fig. 3. The aberrant mitotic division induced by TTFields disrupts normal cell cycle progression, thereby inhibiting cellular proliferation^29^. This disruption is largely attributed to TTFields’ ability to interfere with microtubule polymerization, a crucial process in the formation of the mitotic spindle, which consequently obstructs the proper segregation of chromosomes during mitosis^9,30^. In addition to disrupting the internal structures of cancer cells, TTFields also modulate the dynamics of the cytoskeleton, which is vital for cellular motility. The changes in cytoskeletal, combined with the direct effect on the actin-myosin apparatus, impair the cancer cells’ ability to adhere and migrate efficiently^13,31^. As a result, TTFields significantly diminish the migratory potential of BTC cells, which is a characteristic of aggressive tumor behavior.

The tumor microenvironment (TME) of BTC is characterized by immunosuppression, featuring extensive infiltration of myeloid-derived suppressor cells (MDSCs) and regulatory T cells (Tregs), coupled with abundant inhibitory checkpoint molecules that collectively establish an immunologically “cold” phenotype^32–34^. The TTFields-induced release of ICD markers—including cell surface-translocated CRT, secreted HMGB1, and extracellular ATP—represents a critical mechanism for immune system activation and recognition. Surface-exposed CRT functions as a phagocytic “eat-me” signal that facilitates dendritic cell (DC) uptake of dying tumor cells, while HMGB1 and ATP serve as potent stimulators of DC maturation and enhanced antigen presentation capacity, respectively^35,36^. This coordinated release of danger signals suggests that TTFields treatment may facilitate the transformation of the characteristically immunosuppressive BTC microenvironment from a “cold” to a more immunologically active “hot” phenotype, potentially enhancing the efficacy of concurrent immunotherapeutic interventions.

The combination of TTFields with ICIs such as anti-PD-1 or anti-PD-L1 antibodies has been explored in several preclinical models and clinical trials. For example, a separate study involving a mouse lung tumor model revealed that the application of TTFields in conjunction with ICIs significantly diminished tumor size. Notably, in groups receiving TTFields alongside anti-PD-1/anti-CTLA-4 or anti-PD-L1, there was a pronounced increase in immune cell infiltration, particularly cytotoxic T cells, into the tumor microenvironment^20^. Additionally, a recent study by Nitta et al. revealed that the concurrent administration of anti-PD-1 and TTFields in a GBM mouse model elicited robust myeloid and T-cell-mediated anti-tumor responses, leading to improved survival rates. This result suggests that TTFields treatment may facilitate the transformation of GBM into an “immunologically hot” tumor, thereby making it a more appealing target for immunotherapeutic approaches^37^. In the groundbreaking 2-THE-TOP clinical trial conducted by Novocure, the combined adjuvant treatment of temozolomide, TTFields and pembrolizumab has demonstrated promising results in patients with newly diagnosed glioblastoma. This innovative triplet therapy significantly improved PFS and OS compared to historical controls, highlighting the potential of integrating TTFields with ICIs^38^.

Our findings demonstrate that TTFields upregulate ICD biomarkers in BTC cells, providing a theoretical foundation for the combination of TTFields with ICIs in BTC therapy. Although these results are encouraging, a limitation of this study is that both tumor cell lines used were derived from Asian patients, which limits the generalizability of our findings. Another limitation is the absence of appropriate BTC mouse models, restricting our ability to assess the in vivo immunomodulatory effects of TTFields within the tumor microenvironment. Future studies should incorporate functional immune assays, including dendritic cell phagocytosis assays, to comprehensively assess TTFields’ effects on antigen presentation and immune activation. Additionally, developing BTC-specific animal models will be essential to validate these in vitro findings and evaluate the long-term efficacy of TTFields-ICI combination therapy.

In summary, the application of TTFields represents an innovative therapeutic approach for cancer treatment. The ability of TTFields to induce ICD biomarkers presents a unique opportunity to merge physical interventions with immunomodulatory agents, thereby creating new pathways for the management of BTC.

## Materials and methods

### Cell culture

Human intrahepatic cholangiocarcinoma cell lines HCCC-9810 and RBE, both derived from Asian patients, were purchased from the Type Culture Collection of the Chinese Academy of Sciences. The cells were cultured in RPMI-1640 (Gibco, Grand Island, NY, USA) supplemented with 1% penicillin/streptomycin (pen/strep) (Gibco) and 10% heat-inactivated fetal bovine serum (FBS) (Gibco). All cell lines were maintained in a humidified chamber containing 5% CO_2_ at 37 °C.

## TTFields application in vitro

HCCC-9810 cells (3 × 10^4^) or RBE cells (2 × 10^4^) were seeded on 20 mm cell culture slides and cultured for 24–72 h, and then the slides were transferred to 100 mm cell culture dishes (one slides/dish) using sterile forceps. For the control group (no electric field exposure), the culture dishes were placed directly into a standard incubator for conventional culture. The results of the preliminary experiment showed that there was no statistically significant difference in the number of cells between the control group under routine cultivation and the sham group, which was placed in the TTFields device without electric field exposure (Supplementary Fig. 4). In the TTFields-treated group, cells on the slides were subjected to an alternating electric field at a strength of 1–3 V/cm and a frequency of 100–250 kHz, generated by a TTFields delivery system (Healthy Life Innovation Medical Technology, Jangsu, China). In brief, two pairs of insulated electrodes consisting of high dielectric constant ceramic sheets (ε > 3000) were inserted vertically into the cell culture dish. The electrodes were connected to sinusoidal electric potential waveform generator generating fields of the desired frequencies in the medium. The orientation of the TTFields were switched approximately 90° every 1 s. The transmission of the electric field generates a non-negligible amount of heat inside the Petri dish, dependent on the intensity of the applied field. The device with the petri dish attached is placed in an incubator and the temperature inside the petri dish is maintained at 36.5–37.5 °C throughout the experiment by adjusting the temperature of the incubator. The temperature was measured by 4 thermistors attached to the ceramic walls. At the end of the treatment, the number of cells was determined using the Scepter 3.0 (Merck Millipore, Billerica, Massachusetts) automated cell counter. The relative number of cells at the end of treatment was expressed as percentage of untreated control.

To ensure that the applied electric field intensity corresponded to the expected values, custom-designed electrodes were inserted into the culture dish, and measurements were carried out using a Tektronix MDO4024C oscilloscope. The measured voltage values were then converted to the effective electric field strength. The effective electric field strength (E) was calculated as E = V_pp_/2$\:\sqrt{2}$d, where V_pp_ is the measured peak-to-peak voltage and d is the distance between the probes distance.

### Trypan blue exclusion assay

Cell viability was determined using the trypan blue exclusion method. BTC cells were harvested by trypsinization (Thermo Fisher Scientific, Waltham, MA, USA), collected by centrifugation, and washed twice with PBS (Solarbio). The cell pellet was resuspended in PBS to obtain a single-cell suspension. Equal volumes of cell suspension and 0.4% trypan blue solution (Sigma-Aldrich, St. Louis, MO, USA) were mixed and incubated for 3 min at room temperature. Following incubation, 10 µL of the cell-dye mixture was loaded into the counting chamber of a Countess 3 FL Automated Cell Counter (Thermo Fisher Scientific). The instrument automatically distinguished and enumerated viable cells (trypan blue-negative, unstained) and non-viable cells (trypan blue-positive, blue-stained). Cell viability was expressed as the percentage of viable cells relative to the total cell count, and cell mortality was calculated as 100% minus the viability percentage.

### Colony formation assay

TTFields-treated HCCC-9810 and RBE cells (3000 cells/well) were inoculated into 6-well plates (WHB, Shanghai, China) for overnight growth, followed by change of cell culture medium every two days. After 7–14 days, formed colonies (cluster of 50 cells or more) were fixed with 4% formaldehyde (Solarbio, Beijing, China) throughout 30 min incubation at room temperature and then were stained with 1% Crystal Violet Solution (Beyotime Biotechnology, Shanghai, China) for 10 min. To eliminate the background staining the cells were washed twice with ddH_2_O. Colonies were photographed then counted with the ImageJ version 1.52a (Bethesda, MD, USA) software (http://imagej.nih.gov/ij).

### Wound‑healing assay

The BTC cells (1.5 × 10^5^) were seeded in cell culture slides, and subsequently grown to 75–80% confluency. A sterile 200 µl pipette tip was then used make a wound in the cells, washed the detached cells with phosphate buffer solution (PBS; Solarbio), and the medium was replaced with serum‑free medium. The attached cells were further incubated with TTFields at 2.1 V/cm for 24–72 h. Cell migration into the wound area was observed and photographed using an optical microscope (Olympus, Tokyo, Japan) at 0, 24–72 h. The cell migration rate was calculated using the following formula: Cell migration rate (%) = (Original Area - Area after migration) × 100/Original Area.

### Immunofluorescence

For spindle structure analysis, HCCC-9810 and RBE cells were grown on glass culture slides and treated using the TTFields system for 96 h. Cells were fixed with 4% paraformaldehyde (PFA; Solarbio) for 15 min, permeabilized in PBS containing 0.1% Triton X-100 and blocked with 1% bovine serum albumin (BSA; Beyotime Biotechnology) in PBS for 1 h at room temperature. The cells were stained with rabbit anti-human α-tubulin antibodies (1:800; ab52866; Abcam; Cambridge, UK) overnight at 4 °C. Next day, cells were then incubated with the secondary antibodies Alexa Fluor 488-conjugated anti-rabbit antibody (1:1000; ab150081; Abcam; Cambridge, UK) at room temperature for 1 h in the dark. Nuclei was stained with the dye 4′,6-diamidino-2-phenylindole (DAPI) (Solarbio) at 0.2 µg/ml for 10 min. Fluorescent images were captured with an Olympus IX73 fluorescence microscope (Olympus, Tokyo, Japan).

### LDH assay

LDH activity was measured using the LDH Cytotoxicity Assay Kit following the manufacturer protocol (Beyotime Biotechnology). Briefly, all cells were incubated at 5% CO_2_, 90% humidity and 37˚C for 24 h and then treated with control or 2.1 V/cm TTFields for 96 h. Subsequently, 120 µl supernatant per well was carefully transferred into the corresponding wells of a clear 96‑well plate. Add a total of 60 µl of reaction mixture to each well and incubate for 30 min at room temperature (~ 25 °C) away from light. Absorbance was measured at 490 nm on a Tecan Spark^®^ 20 M (Tecan, Mannedorf, Switzerland). Cytotoxicity (LDH release rate) was calculated according to the following formula: Cytotoxicity (%) = (test sample - blank control) × 100/(maximum enzyme activity control ‑ blank control).

### Calreticulin cell surface expression assays

HCCC-9810 and RBE cells were collected and stained with rabbit polyclonal anti-calreticulin antibody (1:100; ab92516; Abcam; Cambridge, UK) or isotype control rabbit IgG (1:100; ab172730; Abcam; Cambridge, UK) in flow cytometry buffer (1% BSA in PBS) for 45 min. Cells were then washed and incubated with anti-rabbit Alexa Fluor 488 conjugated antibody (1:2000; ab150081; Abcam; Cambridge, UK) in flow cytometry buffer for 30 min. Finally add 500 µL of Propidium Iodide (PI) staining working solution (1:500; abs9358; absin; Shanghai, China) and incubate at room temperature for 15 min. Labeled cells were subsequently detected using a BD Accuri C6 flow cytometer (BD Biosciences, San Jose, CA, USA). Data were analyzed using FlowJo Software version 10 (TreeStar, San Francisco, CA, USA).

### ATP and HMGB1 assays

Supernatants from HCCC-9810 and RBE cells treated with TTFields for 96 h were collected for HMGB1 and ATP release assays. ATP concentrations in cell supernatants were measured by an enhanced ATP assay kit (Beyotime Biotechnology) according to the manufacturer’s instructions. The HMGB1 levels in the supernatant of treated cells were analyzed by an ELISA kit (Meimian, Shanghai, China) according to the manufacturer’s protocol. Luminescence and absorbance were measured using a Tecan Spark^®^ 20 M microplate multimode reader (Tecan, Mannedorf, Switzerland).

### Statistical analysis

Data are presented as mean ± standard deviation (SD). Normality was assessed using the Shapiro-Wilk test, and homogeneity of variance was verified using Brown-Forsythe test. All statistical analyses were performed using GraphPad Prism version 9 (GraphPad Software, Inc., San Diego, CA, USA). For comparisons between two groups, an unpaired two-tailed Student’s *t*-test was applied. For multi-group comparisons, a homogeneity test of variance (Brown-Forsythe) was first performed. One-way ANOVA followed by Bonferroni’s post hoc test was conducted, and if the variance was uneven, a Welch ANOVA was used. *p* < 0.05 was considered statistically significant and indicated as *p < 0.05; **p < 0.01; and ***p < 0.001.

## Supplementary Information

Below is the link to the electronic supplementary material.

Supplementary Material 1

## Data Availability

The datasets generated during and/or analysed during the current study are available from the corresponding author on reasonable request.
