# Supplementary material for: Tumor treating fields suppress tumor cell growth and induce immunogenic cell death biomarkers in biliary tract cancer cell lines
Source: Sci Rep. 2025 Aug 20;15:30611. doi: 10.1038/s41598-025-16341-6 (PMC12368140; doi:10.1038/s41598-025-16341-6)
Supplement: Supplementary file 1 — Supplementary Material 1 [file 41598_2025_16341_MOESM1_ESM.docx]

**Tumor Treating Fields Suppress Tumor Cell Growth and Induce Immunogenic Cell Death Biomarkers in Biliary Tract Cancer Cell Lines**

Ying Yue^1^, Yingying Wang^1^, Jingjing Feng^1^, Min Yao^1^, Yuanzhen Suo^1,2^*

^1^Healthy Life Innovation Medical Technology Co., Ltd, Wuxi 214174, China

^2^Liangzhu Laboratory, Zhejiang University, Hangzhou 310058, China

*Correspondence to: Yuanzhen Suo (suoyuanzhen@zju.edu.cn)

Supplementary Figure 1 Absolute cell counts. HCCC-9810 and RBE cells were treated with TTFields (2.1 V/cm) for 96 h and cell counts were determined (n=6, ****p*<0.001).


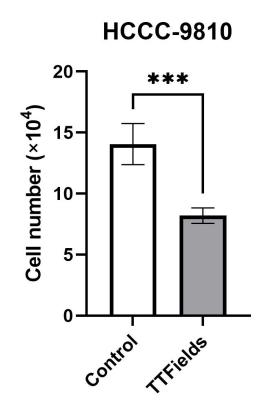

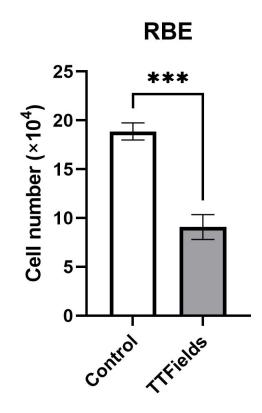


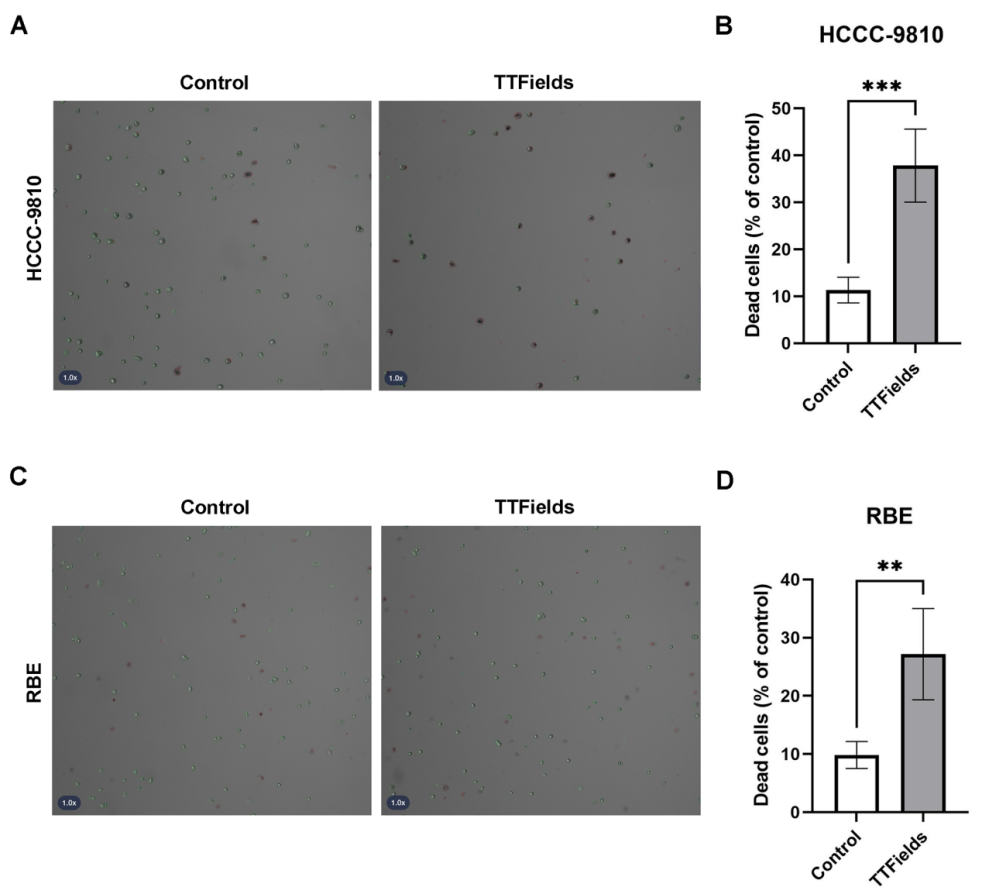


Supplementary Figure 2. Trypan blue staining of BTC cells. (A) Representative images of HCCC-9810 cells stained with trypan blue. Dead cells (stained blue) are indicated by red circles. (B) Percentage of dead cells in the HCCC-9810 cell population following 96 h treatment with 2.1 V/cm TTFields (n=6, ****p*<0.001). (C) Representative images of RBE cells stained with trypan blue. Dead cells (stained blue) are indicated by red circles. (D) Percentage of dead cells in the RBE cell population following 96 h treatment with 2.1 V/cm TTFields (n=6, ***p*=0.002).

**
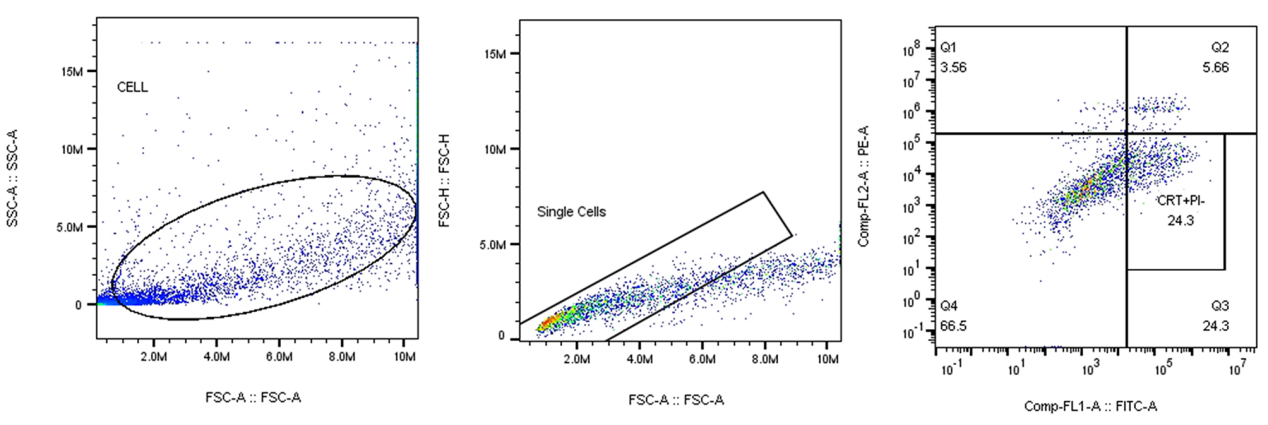
**

Supplementary Figure 3 Gating Strategy.


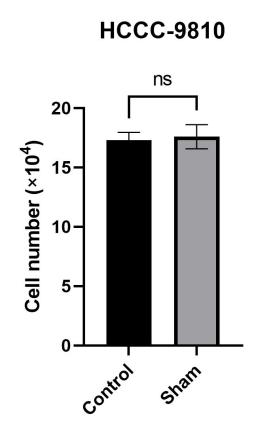

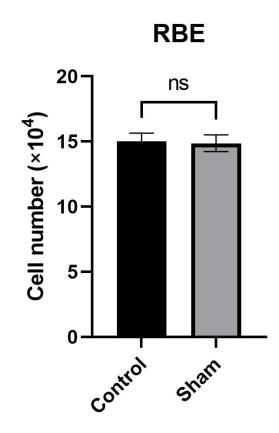


Supplementary Figure 4 Absolute cell counts of Control group and Sham group. (Control group: no treat for 96 h, Sham group: placed in the electric field device without applying an electric field for 96 h, ns.*p*>0.05).
